# Unique patterns of lower respiratory tract microbiota are associated with inflammation and hospital mortality in acute respiratory distress syndrome

**DOI:** 10.1186/s12931-019-1203-y

**Published:** 2019-11-06

**Authors:** Michihito Kyo, Keisuke Nishioka, Takaaki Nakaya, Yoshiko Kida, Yuko Tanabe, Shinichiro Ohshimo, Nobuaki Shime

**Affiliations:** 1 Department of Emergency and Critical Care Medicine, Graduate School of Biomedical and Health Sciences, Hiroshima University, 1-2-3 Kasumi, Minami-ku, Hiroshima, 734-8551 Japan; 2 Department of Infectious Diseases, Kyoto Prefectural University of Medicine, 465 Kajii-cho, Kawaramachi-Hirokoji, Kamigyo-ku, Kyoto, 602-8566 Japan

**Keywords:** Lung, 16S rRNA, Pneumonia, Sepsis, Acute respiratory distress syndrome, Bronchoalveolar lavage

## Abstract

**Background:**

The lung microbiome maintains the homeostasis of the immune system within the lungs. In acute respiratory distress syndrome (ARDS), the lung microbiome is enriched with gut-derived bacteria; however, the specific microbiome associated with morbidity and mortality in patients with ARDS remains unclear. This study investigated the specific patterns of the lung microbiome that are correlated with mortality in ARDS patients.

**Methods:**

We analyzed the lung microbiome from the bronchoalveolar lavage fluid (BALF) of patients with ARDS and control subjects. We measured the copy numbers of 16S rRNA and the serum and BALF cytokines (interleukin [IL]-6, IL-8, receptor for advanced glycation end products, and angiopoietin-2).

**Results:**

We analyzed 47 mechanically ventilated patients diagnosed with (*n* = 40) or without (*n* = 7; control) ARDS. The alpha diversity was significantly decreased in ARDS patients compared with that of the controls (6.24 vs. 8.07, *P* = 0.03). The 16S rRNA gene copy numbers tended to be increased in the ARDS group compared with the controls (3.83 × 10^6^ vs. 1.01 × 10^5^ copies/mL, *P* = 0.06). ARDS patients were subdivided into the hospital survivor (*n* = 24) and non-survivor groups (*n* = 16). Serum IL-6 levels were significantly higher in the non-survivors than in the survivors (567 vs. 214 pg/mL, *P* = 0.027). The 16S rRNA copy number was significantly correlated with serum IL-6 levels in non-survivors (r = 0.615, *P* < 0.05). The copy numbers and relative abundance of betaproteobacteria were significantly lower in the non-survivors than in the survivors (713 vs. 7812, *P* = 0.012; 1.22% vs. 0.08%, *P* = 0.02, respectively). Conversely, the copy numbers of *Staphylococcus*, *Streptococcus* and Enterobacteriaceae were significantly correlated with serum IL-6 levels in the non-survivors (r = 0.579, *P* < 0.05; r = 0.604, *P* < 0.05; r = 0.588, *P* < 0.05, respectively).

**Conclusions:**

The lung bacterial burden tended to be increased, and the alpha diversity was significantly decreased in ARDS patients. The decreased Betaproteobacteria and increased *Staphylococcus*, *Streptococcus* and Enterobacteriaceae might represent a unique microbial community structure correlated with increased serum IL-6 and hospital mortality.

**Trial registration:**

The institutional review boards of Hiroshima University (Trial registration: E-447-4, registered 16 October 2019) and Kyoto Prefectural University of Medicine (Trial registration: ERB-C-973, registered 19 October 2017) approved an opt-out method of informed consent.

## Introduction

Acute respiratory distress syndrome (ARDS) is a fatal disease that causes severe injury to alveolar epithelial cells and subsequent severe respiratory failure due to lung fibrosis, which results in high mortality rates of up to 40%. Approximately 10% of patients admitted to intensive care units (ICUs) are reported to have ARDS [1]. Therefore, ARDS is one of the most severe problems in the intensive care setting; its onset mechanisms must be elucidated, and novel therapeutic methods must be developed.

New methods have enabled analyzing the respiratory microbiota that cannot be described by conventional culture methods [2–5]. Consequently, the respiratory microbiotas have been reported in patients with various respiratory diseases, such as acute exacerbation of interstitial pneumonia (IP), chronic obstructive pulmonary disease (COPD) and cystic fibrosis (CF) [6–10]. In ARDS patients with sepsis, bacteria in the gastrointestinal tract become enriched in the lower respiratory tract (LRT) [11], suggesting that interactions occur between the LRT and the gastrointestinal tract. In addition, analysis of the lung microbiota in patients with trauma showed that ARDS occurrence was associated with increased *Enterobacteriaceae* [12]. Formation of the lung microbiota is also involved in the immune response [13–15]. However, the specific microbiome in the LRT related to morbidity and mortality in the LRT of ARDS patients remains unclear.

This study aimed to clarify whether the microbiota in the LRT is associated with ARDS prognosis and the severity of systemic inflammation by using next-generation sequencing, estimating the bacterial load in the bronchoalveolar lavage fluid (BALF), and measuring the serum and BALF cytokine levels.

## Materials and methods

### Subjects

We studied patients who were intubated and mechanically-ventilated and performed bronchoalveolar lavage (BAL) for diagnostic purposes in the ICU of Hiroshima University Hospital between March 2016 and February 2018. ARDS was clinically defined using the Berlin criteria [16]. Patients younger than 18 years were excluded. The institutional review boards of Hiroshima University (Trial registration: E-447, registered 5 August 2016) and Kyoto Prefectural University of Medicine (Trial registration: ERB-C-973, registered 19 October 2017) approved this study protocol, which waived the need for informed consent because this study was observational study and analyze the residual blood and BALF samples which were obtained for clinical diagnosis. Informed consent for analyzing the residual blood and BALF for research was subsequently obtained from patients or their surrogates.

### Data collection

We collected demographic data including age, sex, underlying clinical conditions, Sequential Organ Failure Assessment (SOFA) and Acute Physiology and Chronic Health Evaluation (APACHE) II scores upon ICU admission. We also recorded the treatment received by the ARDS patients and in-hospital mortality.

### Sample collection

BALF was collected to diagnose the cause of the ARDS within 24 h after intubation by instilling 100 to 150 mL of sterile isotonic saline, with gentle suctioning. BALF samples were immediately stored at − 80 °C. Blood was collected within 24 h after obtaining the BALF. Blood samples were centrifuged, and the serum was immediately stored at − 80 °C.

### 16S rRNA gene amplicon sequencing using NGS

BALF pellets were obtained, and bacterial DNA was extracted via centrifugation at 6000 rpm for 5 min at 4 °C, using NucleoSpin Microbial DNA (Macherey-Nagel, Düren, Germany). Extracted DNA was subjected to PCR to amplify the V5–V6 region of the 16S rRNA gene using specific primers via next-generation sequencing (NGS) with the 784F and NGS.1061R primers, including the adaptor and barcode sequences for NGS (Additional file 1: Table S1). The PCR products were size-selected and purified. Sample libraries were prepared, and the NGS was performed per the method proposed by Akiyama K et al. [17], using the Ion One Touch, the Ion OneTouch ES system, and the Ion Torrent Personal Genome Machine (Thermo Fisher Scientific, Waltham, MA, USA).

### NGS data analysis

Reads sequenced to more than 250 base pairs and Phred scores of more than 20 were obtained from FASTQ files, using the open source pipeline, Quantitative Insights Into Microbial Ecology (QIIME), version 1.8.0 [18]. Ten thousand reads per sample were picked randomly using SeqKit [19]. Operational taxonomic units (OTUs) were selected at 97% sequence identity against the Greengenes database (13_8). Alpha and beta diversity analyses (via the Shannon Index and weighted UniFrac) were performed using QIIME.

### Quantification of the bacterial 16S rRNA gene by real-time PCR

The V5–V6 region of the 16S rRNA gene was amplified using PowerUp SYBR Green Master Mix (Thermo Fisher Scientific) with 0.5 μM of each specific primer (Additional file 1: Table S1) in the StepOnePlus Real-Time PCR System (Applied Biosystems, Foster City, CA, USA). The 16S rRNA gene copy number was estimated as the total bacterial amount, and each bacterial class or genus copy number was calculated by multiplying the 16S rRNA gene copy number × the rate indicated by the NGS.

### Cytokine measurement in ARDS and control patients

Interleukin (IL)-6, IL-8, receptor for advanced glycation end products (RAGE) and angiopoietin-2 (Ang2) were measured in the serum and BALF specimens. IL-6, IL-8, RAGE and Ang2 were measured using an enzyme-linked immunosorbent assay (R&D Systems, Inc., MN, USA) following the manufacturer’s protocol.

### Statistical analysis

Data are expressed as the medians (interquartile ranges [IQRs]) or numbers (percentages) as appropriate. Fisher’s exact test, a nonparametric Mann-Whitney *U* test, and Spearman’s rank correlation coefficient were used to compare the differences between the ARDS survivors and non-survivors. Cox regression analysis was performed to assess the contributions of specific bacterial ratios to the prognosis. *P*-values < 0.05 were considered statistically significant. All statistical analyses were performed using JMP statistical software (version 14.0.0; SAS, Cary, NC, USA).

## Results

### Patients demographics

We collected 47 BALF samples, including 40 samples from ARDS patients and 7 from controls without ARDS (Additional file 1: Table S2). We compared the characteristics of hospital survivors (*n* = 24) and non-survivors (*n* = 16). The patients’ median ages, SOFA scores, and APACHE II scores did not significantly differ between the two groups (Table 1), nor did the rates of prior antibiotic use significantly differ (79% vs. 81%).

**Table 1:** ARDS patient characteristics

	ARDS (*N* = 40)	Survivor (*N* = 24)	Non-survivor (*N* = 16)	*p*
Age (year)	67 (59–77)	67 (60–77)	67 (57–80)	0.62
Male gender	28 (70)	17 (71)	11 (69)	1.00
SOFA score at ICU admission	9 (7–11)	9 (7–11)	10 (7–12)	0.98
APACHE II at ICU admission	28 (22–34)	27 (22–35)	31 (23–34)	0.47
ARDS risk factor				0.11
Pneumonia	26 (65)	19 (79)	7 (44)	
Sepsis	9 (23)	3 (13)	6 (38)	
Aspiration	3 (8)	1 (4)	2 (13)	
Other	1 (3)	1 (4)	0 (0)	
Unknown	1 (3)	0 (0)	1 (6)	
ARDS direct, (%)	28 (70)	18 (75)	10 (63)	0.49
Bacterial pneumonia, (%)	10 (25)	7 (29)	3 (19)	0.71
Prior antibiotic use, (%)	32 (80)	19 (79)	13 (81)	1.00
ICU stay, median	15 (7–24)	15 (7–22)	13 (4–28)	0.73
Mechanical ventilation days, median	10 (6–21)	10 (6–19)	9 (4–23)	0.65

Values are given as the median (interquartile range) or number (%). *P*-values were calculated using Fisher’s exact tests or Mann-Whitney *U* tests

*SOFA* Sequential Organ Failure Assessment, *ICU* Intensive Care Unit, *APACHE* Acute Physiology and Chronic Health Evaluation, *ARDS* Acute Respiratory Distress Syndrome

### Decreased diversity in BALF microbiotas in ARDS non-survivors

**Fig. 1:**
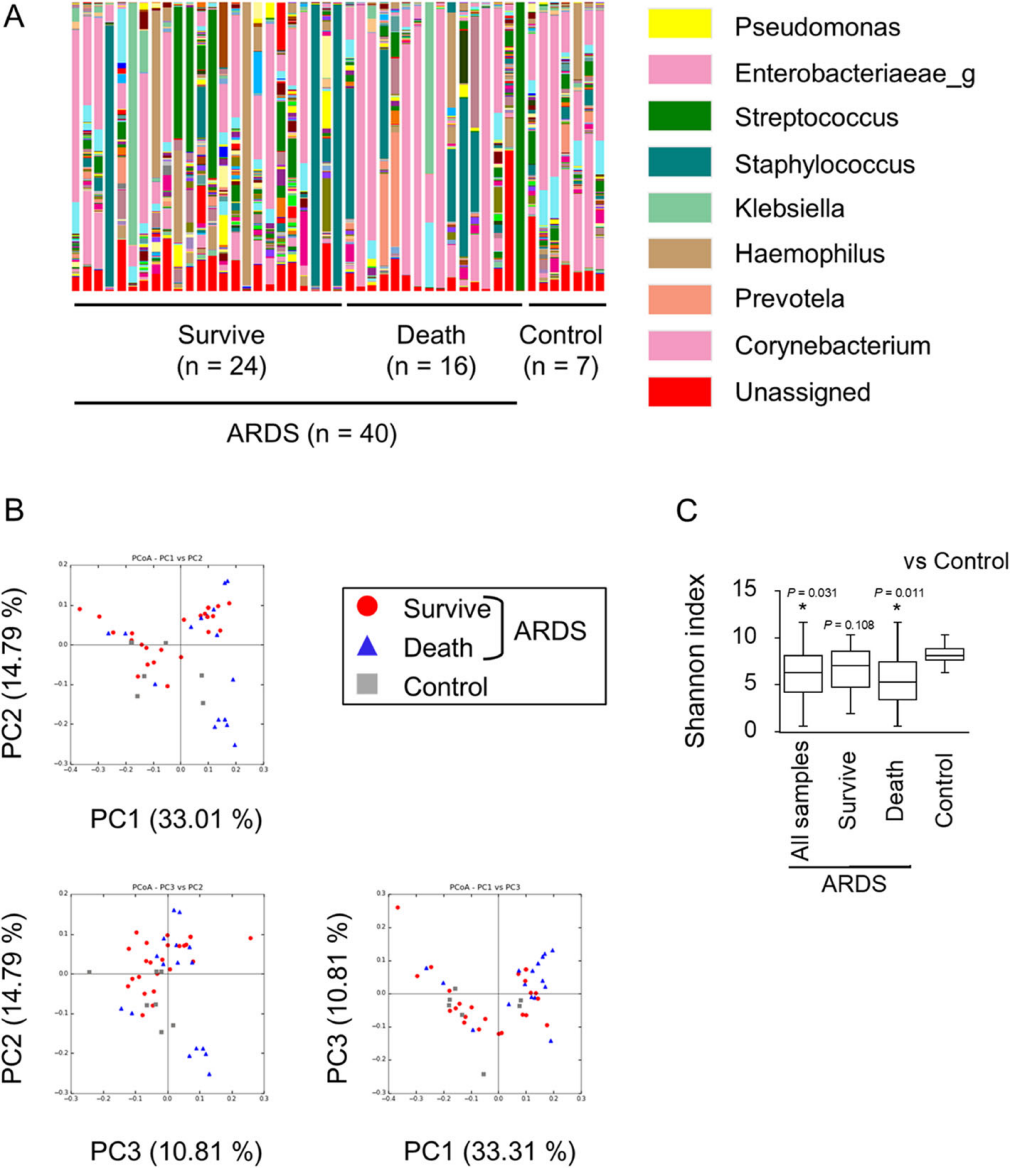
**a** 16S rRNA amplicon sequencing results of the main OTUs at the genus level. The microbiota indicated large variations among individuals. The non-survivor ARDS group presented fewer bacterial genera compared with the survivor and control groups. **b** Microbial community structures of the ARDS patients and controls using principle coordinates analysis. UniFrac distances were calculated after constructing the phylogenetic tree. Each dot and percentage of axes represent one sample and contribution rate, respectively. **c** Comparison of alpha diversity and Shannon diversity index indicating the bacterial diversity within one sample between ARDS patients and controls. Alpha diversities of the ARDS patients and non-surviving ARDS patients were significantly lower than those of the controls (*P < 0.05). ARDS, acute respiratory distress syndrome; OTU, operational taxonomic units

To analyze the microbiota in the BALF, we performed 16S rRNA gene amplicon sequencing using NGS. Figure 1a shows the sequence results and genus level microbial compositions of the ARDS patients and controls (Additional file 2). The dominant bacteria from the sequence results were almost identical to the results of the traditional sputum cultures from patients with bacterial pneumonia (Additional file 1: Table S2). No differences in the microbial community structure were observed between the survivors, non-survivors and controls according to the weighted UniFrac distances using beta diversity analysis (Fig. 1b). Conversely, the alpha diversity analysis, represented by the Shannon index, which shows individual microbiota diversity, showed a significant decrease in the microbiota diversity for all ARDS patients and non-survivors compared with the control group (Fig. 1c). These results indicated that the BALF microbiota was relatively diverse, and BALF microbiota in the ARDS patients showed decreased diversity, which may contribute to ARDS pathophysiology.

### Increased bacterial loads in ARDS patients and higher levels of serum IL-6 in ARDS non-survivors

**Fig. 2:**
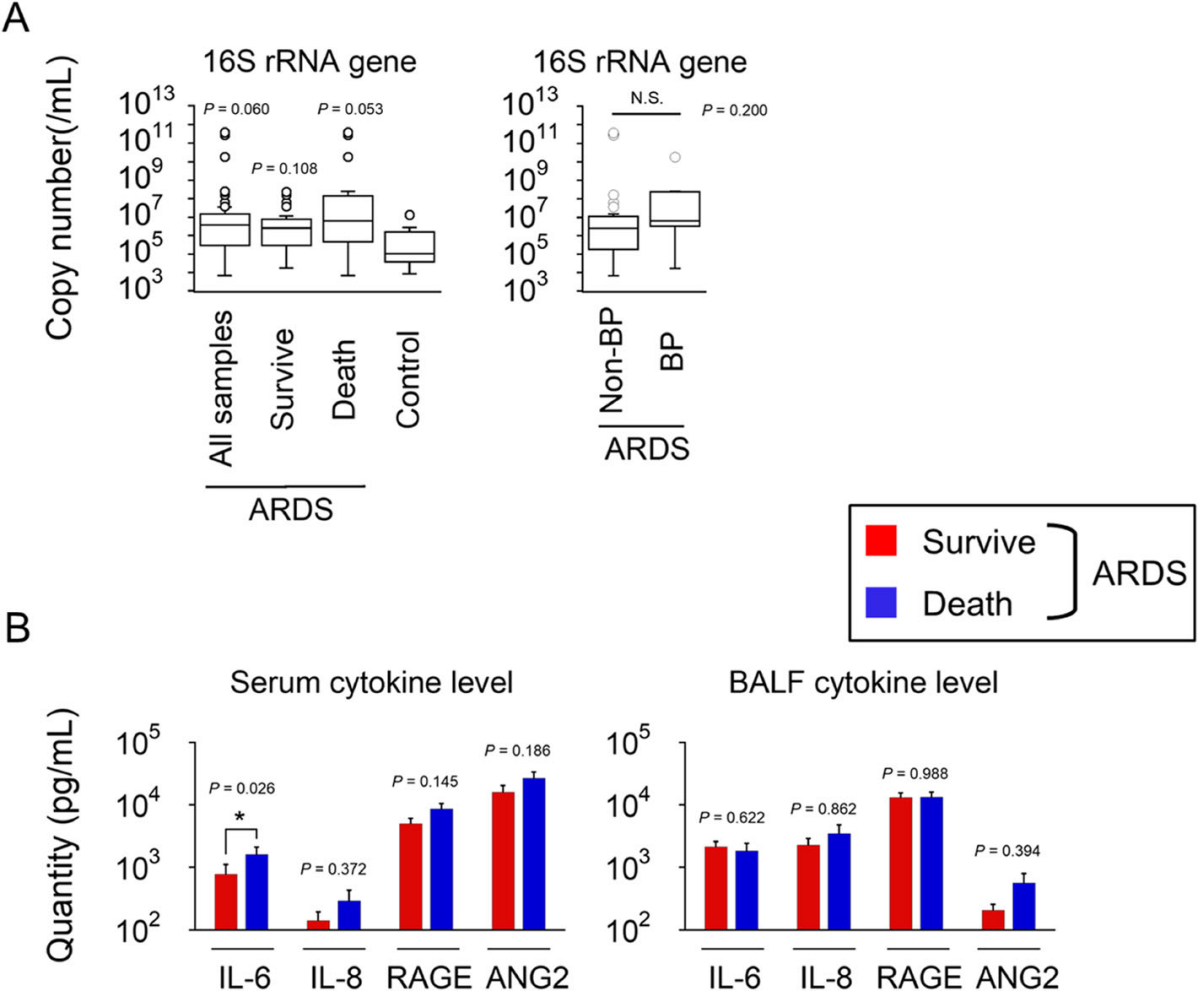
**a** (Left) 16S rRNA copy numbers from the BALF of ARDS patients were increased compared with the controls. (Right) 16S rRNA copy numbers from the BALF did not significantly differ between the BP and non-BP among ARDS. We compared the 10 patients with bacterial pneumonia to 30 patients with non-bacterial pneumonia. **b** Serum and BALF cytokine levels including IL-6, IL-8, RAGE and Ang-2 were compared between surviving and non-surviving ARDS patients. Serum IL-6 levels in 22 survivors were significantly increased compared with those of 13 non-survivors (*P < 0.05). We collected blood samples within 24 h after obtaining the BALF in 35 of ARDS patients. BALF cytokine levels were measured in 23 survivors and 15 non-survivors. ARDS, acute respiratory distress syndrome; BP, bacterial pneumonia; IL, interleukin; RAGE, receptor of advanced glycation end-products; Ang-2, Angiopoietin-2

We hypothesized that bacterial proliferation in the lung was associated with vigorous inflammation in ARDS patients; therefore, we estimated the copy number of the 16S rRNA genes in the BALF and measured the cytokines related to ARDS prognosis. The increased copy numbers of the 16S rRNA differed between the ARDS patients and controls (3.83 × 10^6^ vs. 1.01 × 10^5^ copies/mL, *P* = 0.06) (Fig. 2a, left). Additionally, the 16S rRNA copy numbers did not significantly differ between patients with bacterial pneumonia-induced ARDS and those with non-bacterial pneumonia-induced ARDS (median value: 6.30 × 10^6^ vs. 2.55 × 10^6^ copies/mL, *P* = 0.20) (Fig. 2a, right). These results showed an increased absolute bacterial amount in ARDS patients compared with those of the controls, which was irrelevant to the ARDS origin. These results indicate that increased absolute amounts of bacteria may play an important role in patients with ARDS.

Subsequently, we measured the serum and BALF cytokines (IL-6, IL-8, RAGE and Ang2), which previous studies found to be associated with mortality in ARDS patients. Among these cytokines, only serum IL-6 was significantly increased in the non-survivor group (Fig. 2b). This suggested that aggressive systemic inflammation had occurred in the non-survivors; thus, we focused on IL-6 as a marker of ARDS to predict hospital mortality.

### Correlation between increased 16S rRNA copy numbers and IL-6 expression in non-survivor patients

**Fig. 3:**
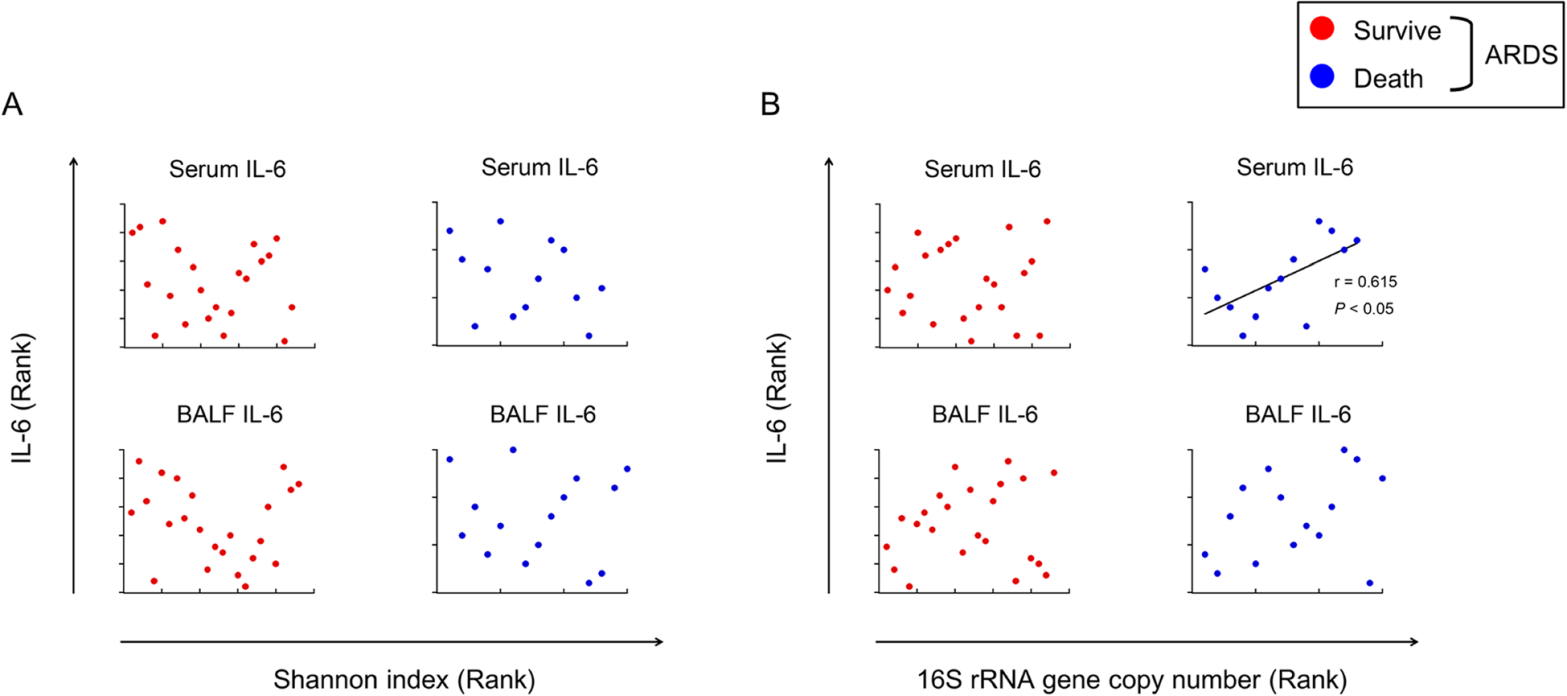
**a** Correlation between alpha diversity (Shannon diversity index) and serum (in 22 survivors and 13 non-survivors) and BALF IL-6 (in 23 survivors and 15 non-survivors). No significant correlation was found between IL-6 and the Shannon diversity index. **b** Correlation between the copy numbers of 16S rRNA genes in the BALF and the serum and BALF IL-6. Increased copy numbers of 16S rRNA were correlated with increased serum IL-6. BALF, bronchoalveolar lavage fluid; IL, interleukin

Because non-survivors may have had aggressive systemic inflammation via higher IL-6 levels, and the Shannon index was decreased and the 16S rRNA copy numbers tended to be increased in ARDS patients compared with the control group, we performed correlation analysis to assess which factors contributed to IL-6 production. An increased Shannon index was not correlated with increased serum or BALF IL-6 concentrations (Fig. 3a), while the 16S rRNA copy number was significantly positively correlated with serum IL-6 concentration in the non-survivor group (Fig. 3b). Thus, increased bacteria in the LRT may correlate with inflammation in non-surviving ARDS patients.

### Unique bacterial pattern in the LRT was correlated with IL-6 in patients with ARDS

**Fig. 4:**
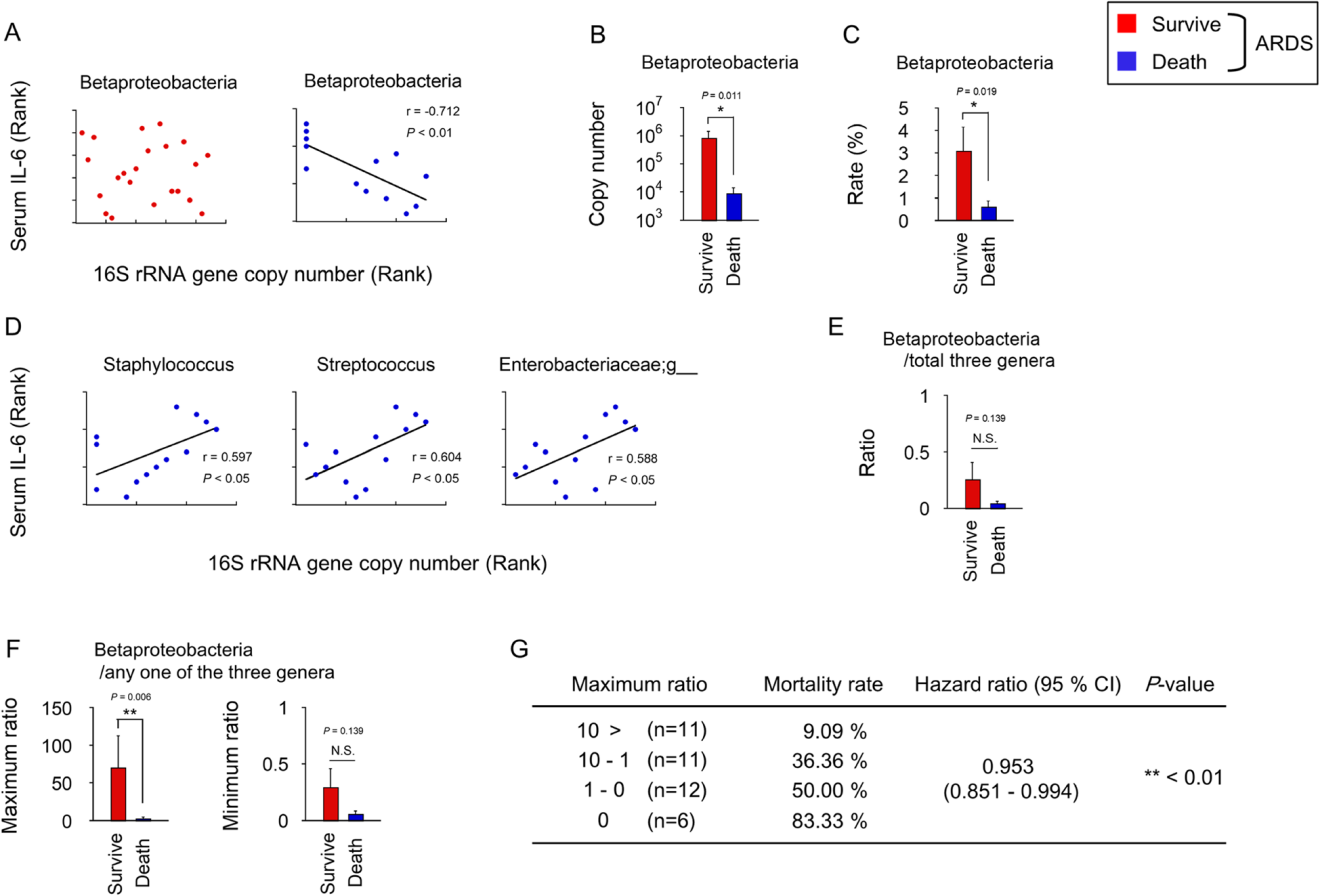
**a** (Left) Copy numbers of the 16S rRNA genes of the Betaproteobacteria were not correlated with serum IL-6 in 22 surviving ARDS patients. (Right) Increased copy numbers of 16S rRNA genes in Betaproteobacteria were correlated with serum IL-6 in 13 non-surviving ARDS patients. **b** Copy numbers of 16S rRNA genes in the Betaproteobacteria in 16 non-surviving ARDS patients were significantly decreased compared with those of 24 surviving ARDS patients (*P < 0.05). **c** The relative abundances of Betaproteobacteria in 16 ARDS non-survivors were significantly decreased compared with those of 24 ARDS survivors (*P < 0.05). **d** Increased copy numbers of 16S rRNA genes of *Staphylococcus, Streptococcus* and Enterobacteriaceae were correlated with increased serum IL-6 levels in 13 non-surviving ARDS patients. **e** The ratio of Betaproteobacteria to *Staphylococcus, Streptococcus* and Enterobacteriaceae did not significantly differ between 24 survivors and 16 non-survivors of ARDS. **f** The ratio of Betaproteobacteria to the maximum value among *Staphylococcus, Streptococcus* and Enterobacteriaceae was significantly decreased in 16 non-survivors of ARDS compared with those of 24 survivors of ARDS (**P < 0.01). **g** Hazard ratio of the ratio of Betaproteobacteria to maximum value among *Staphylococcus, Streptococcus* and Enterobacteriaceae by the Cox proportional hazards model in 40 ARDS patients (**P < 0.01). IL, interleukin; ARDS, acute respiratory distress syndrome

Because no significant correlation was detected in the survivors, specific bacteria likely play an important role in IL-6 expression in non-survivors. Therefore, we searched for characteristic microbiota or bacterial patterns that would indicate a correlation with serum IL-6. The copy number of each bacteria class, order, family and genus was calculated from the results of NGS and 16S rRNA copy number, and then correlation with IL-6 levels was analyzed. We found that one bacterial class, Betaproteobacteria, was strongly negatively correlated (r = − 0.712) with serum IL-6 levels in the non-survivors (Fig. 4a). The copy numbers and relative abundance of the Betaproteobacterial OTUs was significantly decreased in the non-survivors compared with the survivors (Fig. 4b and c). Conversely, *Staphylococcus* and *Streptococcus* at the genus level and Enterobacteriaceae at the family level were significantly positively correlated with serum IL-6 levels in the non-survivors (Fig. 4d). Conversely, these bacteria were not correlated with serum IL-6 concentrations in the surviving ARDS patients (Fig. 4a).

Next, we asked if the ratio of Betaproteobacterial OTUs to the OTUs of *Streptococcus*, *Staphylococcus* and Enterobacteriaceae might be a useful marker for predicting hospital mortality in patients with ARDS. The ratio of the relative abundance of Betaproteobacterial OTUs to the total of the other three OTUs did not significantly differ between the survivor and non-survivor groups (Fig. 4e). Interestingly, the ratio of the relative abundance of Betaproteobacterial OTUs to maximum relative abundance among the other three OTUs was significantly associated with hospital deaths in patients with ARDS (hazard ratio 0.953; 95% CI: 0.851–0.994; *P* = 0.0056) (Fig. 4f, g). This ratio may be a useful marker for predicting hospital mortality in ARDS patients.

## Discussion

In this analysis of the 16S rRNA copy number and the lung microbiome in the BALF via next-generation sequencing, we found three new results. First, the bacterial copy number in the BALF was increased in patients with ARDS compared with that in the control. Second, the Shannon index, which is associated with the microbiota diversity, was significantly lower in patients with ARDS who died during their hospital stay. Third, the quantitative balance among several characteristic bacteria, including Betaproteobacteria, *Staphylococcus*, *Streptococcus* and Enterobacteriaceae, may be involved in the ARDS pathogenesis.

The total bacteria in the LRT in the patients of this study (3 × 10^6^ copies/mL) was greater than that in the healthy controls (10^3^ to 10^4^ copies/mL) [20, 21]. Continuous microaspiration and an impaired natural airway clearance contributed to this increase in patients on mechanical ventilation [22]. Nevertheless, the bacterial copy numbers in the BALF in ARDS patients tended to be increased compared with those of the control patients on mechanical ventilation, even if the number of the control patients were limited. Moreover, bacterial pneumonia, as cause of ARDS, did not affect the bacterial burden. In this study, we judged this difference of the bacterial copy number had an important meaning in the pathogenesis. Increased bacterial loads in the BALF were observed in a lipopolysaccharide-induced mouse model of ARDS [23]. This indicates a possible mechanism for the lung bacterial burden in ARDS patients other than via the respiratory tract. An anaerobic zone caused by inflammation of the alveolar epithelial cells promotes bacterial growth in injured lungs [24]. Our controls included a patient with heart failure; however, alveolar epithelial cells are not typically injured in heart failure. Several studies have indicated that increased lung and gut permeability may induce bacterial migration via gut-draining lymphatics and the portal or systemic circulation [11, 25, 26]. These mechanisms, which occur in the ARDS pathophysiology, could contribute to the increased bacterial numbers and increased morbidity.

Bacterial community diversity in the lungs of critically ill patients on mechanical ventilation has been shown to be lower than that in healthy controls [27]. Our results showed that the Shannon index was significantly lower in ARDS patients than in the controls who were also on mechanical ventilation. This is in line with a previous report showing the Shannon diversity index from BAL samples in a lipopolysaccharide-induced lung injury mouse model were decreased compared with the controls [23]. The balance in bacterial diversity may be disrupted by the markedly increased characteristic bacteria in non-surviving ARDS patients. However, in our results, the Shannon index was not significantly correlated with serum IL-6, a marker of inflammation morbidity.

We showed that the numbers of Betaproteobacteria, which were negatively correlated with IL-6 production, were decreased and that *Staphylococcus, Streptococcus* and Enterobacteriaceae were detected as the characteristic bacteria positively correlated with serum IL-6 in non-surviving ARDS patients. In ARDS patients, the lung microbiota plays an important role in the lung immune system [28]. In addition, a previous animal model showed that introducing bacterial communities from injured lungs into normal lungs provoked more inflammation and injury [23]. *Staphylococcus* and *Streptococcus* were associated with lung inflammation in IP and lung transplant recipients [6, 15, 29]. *Streptococcus* produces pneumolysin and MUC5B, which were correlated with lung cell fibrosis in a mouse model [30] and lung inflammation [31]. *Enterobacteriaceae* played a critical role in ARDS development [11, 12]. In patients who have had a lung transplantation or other respiratory disease, some dominant bacterial communities have been shown to be markers of inflammation and severity [15, 32]. Our results are in line with those of previous studies, suggesting that the balance between pathogenic and commensal bacteria, represented by the ratio of *Staphylococcus, Streptococcus* and Enterobacteriaceae to Betaproteobacteria*,* could become a marker of illness severity in ARDS patients. Moreover, we believe that the link between the lung microbiota and mortality and the integration of the microbiome data with host response assays for ARDS were novel findings. Further investigation is needed to elucidate the detailed mechanisms.

The current study had several limitations. First, this study was a single-center study with a relatively small sample size. Therefore, potential confounders could have influenced the results. Moreover, the number of control patients was limited because performing BAL on patients without respiratory diseases was not ethically acceptable. Second, although analysis of the microbiome in LRT using BALF was reported to be reliable method [5], risk of contamination of the BALF samples should be considered. Generally, when obtaining BALF, passing the upper respiratory tract introduces a major risk of contamination [2]; however, in this study, all patients were intubated, and the BALF was obtained via the intubation tube. Moreover, bacterial contamination via the intubation tube was thought to be minimal because the BALF was obtained within 24 h after intubation. Third, most patients were administered antibiotics before performing the BAL. Antibiotics influence the respiratory tract microbiome [33]; however, administering antibiotics has not been significantly associated with lung community compositions in patients with traumatic ARDS [12]. Our results indicated that the rate of antibiotic administration before BAL did not significantly differ between the hospital survivor and non-survivor groups, and the copy numbers of the microbiome were unaffected by whether antibiotics were administered or not (data not shown). Therefore, the influence of antibiotics is thought to be minimal. Fourth, we had no unventilated controls or ventilated normal lung controls, and the characteristics of the control patients were heterogenous. However, indiscriminately performed BAL for no specific reason is unethical because of its invasiveness.

## Conclusions

In ARDS patients, the lung bacterial burden tended to increase, and the bacterial diversity was significantly decreased compared with that of the controls. Moreover, the unique microbial community structure in ARDS represented by Betaproteobacteria, *Staphylococcus*, *Streptococcus* and Enterobacteriaceae was correlated with increased serum IL-6 levels and hospital mortality. Further investigations with larger sample sizes and uniform characteristics are needed to validate the results of this study.

## Supplementary information

**Additional file 1: Table S1.** Specific primers of the 16S rRNA gene V5–V6 region, including the adaptor and barcode sequences for the next-generation sequencing. **Table S2.** Additional characteristics and information of microorganisms of pneumonia by clinical examination and NGS.

**Additional file 2.** The complete sequences of the QIIME data.

## Data Availability

The datasets used and/or analyzed during the current study are available from the corresponding author on reasonable request.

## Abbreviations

Ang: Angiopoietin
APACHE: Acute Physiology and Chronic Health Evaluation
ARDS: Acute Respiratory Distress Syndrome
BAL: Bronchoalveolar lavage
BALF: Bronchoalveolar lavage fluid
CF: Cystic Fibrosis
COPD: Chronic Obstructive Pulmonary Disease
ICU: intensive care unit
IL: Interleukin
IP: Interstitial pneumonia
LRT: lower respiratory tract
NGS: Next generation sequence
OTU: Operational taxonomic units
QIIME: Quantitative Insights Into Microbial Ecology
RAGE: Receptor of advanced glycation endproducts
SOFA: Sequential Organ Failure Assessment
