# Supplementary material for: Unique patterns of lower respiratory tract microbiota are associated with inflammation and hospital mortality in acute respiratory distress syndrome
Source: Respir Res. 2019 Nov 6;20:246. doi: 10.1186/s12931-019-1203-y (PMC6836399; doi:10.1186/s12931-019-1203-y)
Supplement: Supplementary file 1 — Additional file 1: Table S1. Specific primers of the 16S rRNA gene V5–V6 region, including the adaptor and barcode sequences for the next-generation sequencing. Table S2. Additional characteristics and information of microorganisms of pneumonia by clinical examination and NGS. [file 12931_2019_1203_MOESM1_ESM.docx]

Table S1. Specific primers of the 16S rRNA gene V5–V6 region, including the adaptor and barcode sequences for the next-generation sequencing.


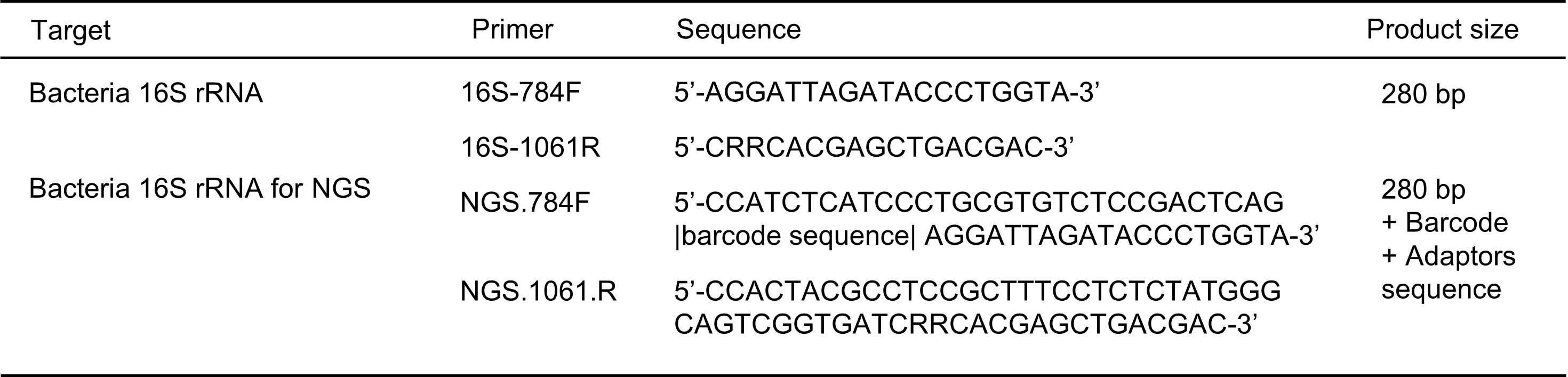


Table S2. Additional characteristics and information of microorganisms of pneumonia by clinical examination and NGS.

| ARDS |  | Microorganism of pneumonia | sputum culture | Dominant bacteria by NGS |
| --- | --- | --- | --- | --- |
| BAL41 | Viral pneumonia | Influenza virus |  |  |
| BAL43 | Viral Pneumonia | Influenza virus |  |  |
| BAL47 | Sepsis |  |  |  |
| BAL48 | Sepsis |  |  |  |
| BAL51 | Pneumonia | Unknown | negative | Enterobacteriaceae |
| BAL55 | Bacterial pneumonia | MSSA | MSSA | Staphylococcus |
| BAL60 | Sepsis |  |  |  |
| BAL63 | Other |  |  |  |
| BAL66 | Bacterial pneumonia | *Klebsiella pneumonia* | *Klebsiella pneumonia* | Klebsiella |
| BAL67 | Sepsis |  |  |  |
| BAL68 | Pneumonia | Unknown | negative | Haemophilus |
| BAL71 | Sepsis |  |  |  |
| BAL72 | Aspiration |  |  |  |
| BAL75 | Pneumonia | Unkonwn | negative | Corynebacterium |
| BAL78 | Viral Pneumonia | Influenza virus |  |  |
| BAL79 | Pneumonia | Unknown | negative | Enterobacteriaceae |
| BAL82 | Aspiration |  |  |  |
| BAL83 | Pneumonia | Unknown | negative | Streptococcus |
| BAL87 | Bacterial pneumonia | *Klebsiella pneumonia* | *Klebsiella pneumonia* | Klebsiella |
| BAL90 | Pneumonia | Unknown | negative | Corynebacterium |
| BAL93 | Sepsis |  |  |  |
| BAL94 | Bacterial pneumonia | *Streptococcus pneumonia* | *Streptococcus pneumonia* | Streptococcus |
| BAL95 | Bacterial pneumonia | *Streptococcus pneumonia* | *Streptococcus pneumonia* | Enhydrobacter |
| BAL100 | Bacterial pneumonia | MRSA | MRSA | Enterobacteriaceae |
| BAL106 | Sepsis |  |  |  |
| BAL108 | Aspiration |  |  |  |
| BAL111 | Unknown |  |  |  |
| BAL112 | Bacterial pneumonia | *Haemophilus influenza* | *Haemophilus influenza* | Haemophilus |
| BAL113 | Viral Pneumonia | Cytomegalovirus |  |  |
| BAL114 | Pneumonia | Unknown | negative | Enterobacteriaceae |
| BAL116 | Sepsis |  |  |  |
| BAL117 | Pneumonia | Unknown | negative | Enterobacteriaceae |
| BAL119 | Pneumonia | Unknown | negative | Streptococcus |
| BAL120 | Sepsis |  |  |  |
| BAL122 | Bacterial pneumonia | *Legionella pneumophila* | *Legionella pneumophila* | Actinomyces |
| BAL125 | Viral Pneumonia | Influenza virus |  |  |
| BAL126 | Bacterial pneumonia | *Streptococcus pneumonia* | *Streptococcus pneumonia* | Streptococcus |
| BAL128 | Viral Pneumonia | Influenza virus |  |  |
| BAL131 | Pneumonia | Unknown | negative | Granulicatella |
| BAL134 | Bacterial pneumonia | MSSA | MSSA | Staphylococcus |
|  |  |  |  |  |
|  |  |  |  |  |
| Controls |  |  |  |  |
| BAL44 | Trauma, VAP s/o |  | negative |  |
| BAL46 | Consciousness disorder, VAP s/o |  | negative |  |
| BAL50 | Trauma, VAP s/o |  | negative |  |
| BAL52 | Aortic dissection, VAP s/o |  | negative |  |
| BAL53 | Trauma, VAP s/o |  | negative |  |
| BAL54 | Consciousness disorder, VAP s/o |  | negative |  |
| BAL86 | Heart failure |  | negative |  |

BAL, bronchoalveolar lavage; NGS, next generation sequence; MSSA, Methicillin Sensitive *Staphylococcus aureus*; MRSA, Methicillin Resistant *Staphylococcus aureus*; VAP, ventilator associated pneumonia.
